# Risk and protective factors associated with the mental health of young adults in Kabul, Afghanistan

**DOI:** 10.1186/s12888-018-1648-4

**Published:** 2018-03-21

**Authors:** Qais Alemi, Carl Stempel, Patrick Marius Koga, Susanne Montgomery, Valerie Smith, Gagandeep Sandhu, Bianca Villegas, Jessica Requejo

**Affiliations:** 1 Department of Social Work & Social Ecology, School of Behavioral Health, Loma Linda University, 1898 Business Center Drive, San Bernardino, CA 92408 USA; 2 Department of Sociology and Social Services, California State University, East Bay, 25800 Carlos Bee Blvd, Hayward, CA 94542 USA; 3 Department of Public Health Sciences, UCD School of Medicine, University of California, One Shields Avenue, Med Sci 1-C, Davis, CA 95616 USA; 4 Department of Health Sciences, California State University, East Bay, 25800 Carlos Bee Blvd, Hayward, CA 94542 USA; 5 Department of Epidemiology, School of Public Health, Loma Linda University, 24951 North Circle Drive, Loma Linda, CA 92350 USA

**Keywords:** Afghanistan, Distress, Hope, Mental health, Resilience, Young adults

## Abstract

**Background:**

We examined the mental health status and severity of psychological distress symptoms among young adults residing in Kabul, Afghanistan and determined how such outcomes might be influenced by an array of risk and protective factors.

**Methods:**

A cross-sectional study design was adopted using convenience, snowball, and street-intercept recruitment techniques. Surveys were completed by 232 young adults between 18 and 35 years of age in September 2015. We used both etic (mental health component of the SF-8) and emic (Afghan Symptom Checklist) measures of mental health and psychological distress, respectively, and regressed these outcome measures against socio-demographic, physical health, and psychological variables (resilience, hope-optimism) using ordinary least squares (OLS) regression methods.

**Results:**

We found that poor mental health is common in this sample, affecting 75% of participants; and, that distress symptoms (depressive, anxiety, and somatoform symptoms) occur often. Regression models were consistent in showing higher education as a risk-factor for both outcomes, whereas, age, ethnicity, and income significantly contributed only to the ASCL model as risk-factors. However, both outcomes were strongly influenced by protective factors such as good physical health status and higher perceived hope-optimism.

**Conclusions:**

Our study provides further evidence of how current economic conditions in Kabul contribute to poor mental health and symptom severity, but also show how positive physical health and perceived hope-optimism can be protective. This study provides support for developing culturally-competent policies and interventions that build on protective factors.

## Background

War has a catastrophic effect on the health and well-being of nations by fragmenting communities and families, disrupting social and economic development, and by bringing long-term physical and psychological harm to children and adults [1]. This is epitomized in Afghanistan, a country ranking among the lowest (169 out of 188 countries) on the human development index [2] and where approximately 17% of the adult population suffers from mental health disorders [3].

Early household-based cross-sectional studies show that the prevalence of depression, anxiety, and post-traumatic stress disorder (PTSD) is high, with female gender and exposure to traumatic events being important risk-factors [4, 5]. More recent studies show that mental health problems for Afghans are not solely rooted in traumatic experiences. Rather, they are largely influenced by poor living conditions associated with a broken economy and poor governance [6]. Among their sample, Miller and Rasmussen [7] show that less than 15% of the variance in levels of PTSD symptoms are accounted for by war exposure, while a significantly larger proportion is accounted for by ongoing daily stressors associated with unmet basic needs. Similar trends are observed among representative samples of children across various municipalities in Afghanistan, where a major risk-factor identified was residence in Kabul, described by the study’s authors as a volatile setting where overcrowding, high living costs, and widening inequalities exacerbate war-related adversities [8].

Due to these conditions, many young people view Afghanistan’s social atmosphere profoundly dispiriting and have become deeply cynical as a result [9], which explains the unprecedented exodus of young people seeking asylum in European nations [10]. Panter-Brick et al.’s [11] survey of young educated adults in Kabul shows that they are not only distressed, but disillusioned by conditions that prevent their social advancement. Surprisingly, however, Panter-Brick and colleagues find that the vast majority of young adults in their sample demonstrate a high degree of hope and fortitude about the future by endorsing the optimistic beliefs that socio-political conditions would improve (74.8%) and that they can be resilient (81.9%), despite adversity. The authors go on to describe young adults in Afghanistan as significant agents of change and economic power for the future of their country.

While these studies are informative, there is a paucity of research on the mental health of young adults in conflict-affected countries like Afghanistan. Patel et al. [12] indicate that a high global burden of mental health in young people is influenced by risk and protective factors within biological, psychological and social domains. The important public health implication of this, according to Patel et al. is that early interventions could reduce symptom severity and prevent comorbidity.

To this end, the present study examines the mental health of young adults between 18 and 35 years of age residing in Kabul, a setting that typifies Galea et al.’s [13] characterization of adverse urban environments known for concentrated disadvantage, crime, and a myriad of other factors that perpetuate chronic psychological and physiological stress. The aim of our work was to further current knowledge of the risk and protective factors affecting the mental health of young adults by exploring two distinct mental health outcome variables. The first is the mental health subscale from the *Short-Form/SF-8* [14], an etic scale which provides an overall assessment of mental health status; and, the other an emic scale of culturally-salient idioms and indicators of distress symptoms developed in Afghanistan, the *Afghan Symptom Checklist* (*ASCL*) [15]. Rasmussen et al. [16] recently examined the value of using this locally-developed emic measure versus an internationally-developed etic measure, the Self-Reporting Questionnaire-20 and found that the ASCL was a better measure of distress symptoms for women (given the ASCL’s ability to tap into local conceptions of distress) while the two measures were similar for men. Here we intend to use etic and emic measures for providing a comprehensive account of mental health, which is conceptually in line with Kabul-based studies by Miller et al. [6] and Panter-Brick et al. [11].

Though compared to Panter-Brick et al.’s study, which focused solely on college educated young adults, our study sample varies by education, allowing us to test previous findings about educated young Afghans by comparing them to less educated Afghans. Our study also advances knowledge of protective factors for mental health, for example, how one’s physical health, never-before-tested measures of individual resilience, as well as culturally-salient coping strategies such as hope and optimism operate in terms of mitigating mental health problems. As informed by previous studies conducted in Afghanistan, we theorize that poor outcomes on both measures will be determined by risk-factors such as female gender, not being married, higher education, unemployment, and unstable income; whereas, good physical health status, as well as psychological factors including measures of resilience and of hope and optimism will exert a protective effect on these outcomes. While these expected associations may be common in other regions, the significance of understanding them in this setting is highly pressing given that young adults are a burgeoning working-age population [17] who enter a job market that provides little hope for upward mobility amid a socio-political climate that does not ensure safety—consequently placing more and more people at risk of mental health problems. Young people are vital to Afghanistan’s peace and prosperity, and unfortunately, current conditions are forcing many to seek asylum at unprecedented rates in western Europe [18], and among those who choose not to exit the country, growing numbers are joining the insurgency solely to earn an income, and engaging in other subversive behaviors such as drug trafficking [19]. Therefore, understanding mental health risk and protective factors in this setting is a starting point for mobilizing policy-makers and public health authorities alike to take action through promoting community-based programs that reduce symptoms, build on their resilience, and protective cultural assets.

## Methods

### Sample, setting, & procedures

In a cross-sectional design, participants were recruited in Kabul in September 2015 using a combination of convenience, snowball, and street-intercept techniques where two data collectors solicited participants with an information sheet providing details about the study, their rights, and the survey. Interestingly, an overwhelming number of individuals asked whether their participation would benefit them in any way with regard to their open immigration cases to the United States (U.S.), and in response we clearly explained that it would not. Upon gaining verbal consent, surveys were completed mainly through self-administration (wherever they were recruited, e.g. on the street, public venues, their places of employment), which took approximately 15 min for participants to complete, while some individuals with literacy challenges were interviewed, which usually lasted approximately 30 min. As a token of our appreciation for their time and in consideration of their real economic needs, participants were offered an equivalent of $10 for completing surveys.

### Instrumentation

#### Dependent variables

##### Mental Health Status

As mentioned, two distinct measures of mental health were used to test our hypotheses. One of these scales included the mental health component of the *SF-8* scale. This scale contains four items assessing: vitality (energy level), social functioning (ability to perform social activities with family or friends when facing physical health or emotional problems), role emotional (ability to perform work, school, or other daily activities when facing personal or emotional problems), and mental health (extent of emotional problems such as anxiety, depression, or irritability) [14]. Items are based on a four-week recall period asking participants to respond using a five or six-point ordinal set of response choices. A norm-based scoring procedure is used in order to generate Mental Component Summary (MCS) scores as indicated in the instrument’s scoring guidelines [20]. Higher scores indicate better mental health status with a score of 50 representing the average of the general U.S. population. While U.S. normative scores provide a useful benchmark to compare our findings, Roberts et al.’s [21] study testing the psychometric properties of the SF-8 among northern Ugandans assists in contextualizing MCS scores with a conflict-affected population, which the authors report MCS scores (*M* = 39.27, *SD* = 12.83) to be much lower than the U.S. normative score of 50. The mental health subscale of the SF-8 demonstrated marginal scale reliability (*Cronbach’s α* = 0.66) in our sample.

##### Psychological Distress Symptoms

The second measure used was a culturally-grounded measure of mental health developed and validated in Afghanistan, the *Afghan Symptom Checklist* (ASCL) [15]. The ASCL’s 23 items include local idioms of distress (e.g. *‘jigar khun’*/sadness due to interpersonal loss, *‘fishar bala’*/internal agitation) as well as symptoms known to western psychiatry relating to depression (e.g. sadness, appetite loss) and PTSD (e.g. nightmares). Items are based on a two-week recall period and are rated on an ordinal five-point scale of 1 (“never”) to 5 (“every day”) with scores ranging from 23 to 115 (higher scores indicative of higher distress). The ASCL demonstrated excellent reliability in this sample (Cronbach’s *α =* .914).

#### Independent Variables

##### Socio-demographic Characteristics

Surveys assessed a number of socio-demographic characteristics such as age, gender, marital status, and ethnicity. Socio-economic characteristics included education (highest level completed ranging from ‘less than high school’ to ‘university degree’), employment status (‘unemployed’, ‘employed’), stability in income (‘yes-no’ question asking participants whether they are comfortable in covering monthly household expenses with current income).

##### Physical Health Status

We used the four items from the physical health component of the SF-8 [14] to assess physical health status. Items on the physical health component relate to physical functioning as related to performing usual physical activities (walking and climbing stairs), role limitations due to physical health problems, bodily pain, and perceived general health. Items are based on a four-week recall period with each item consisting of a five or six-point response choice. Similar to the MCS score described above, a norm based scoring procedure is used in order to generate a Physical Component Summary (PCS) score. Higher PCS scores indicate better physical health status. Among conflict-affected Ugandans [21], PCS scores were considerably lower (*M* = 42.21, *SD* = 11.93) than U.S. normative scores of 50. The PCS subscale demonstrated acceptable reliability in our sample (*Cronbach’s α* = 0.70).

##### Resilience

We measured resilience using the Connor-Davidson Resilience Scale (CD-RISC-25) [22]. The CD-RISC-25 includes items that reflect the ability to tolerate experiences such as change, personal problems, illness, painful feelings, etc. Items are based on a one-month recall period and are rated on a scale of 0 (“not true at all”) to 4 (“true nearly all the time”). Scores are an equally weighted sum of 25 items that range from 0 to 100 with higher scores being indicative of higher resilience. The CD-RISC-25 demonstrated good reliability in this sample (Cronbach’s *α* = .865).

##### Hope and optimism

For brevity, we assessed *perceived hope* using a single-item measure asking participants whether they believed that they ‘would have a better future in Afghanistan as compared to their parents’ with a binary ‘yes-no’ response choice. Similarly, *perceived optimism* was measured using a single item measure asking participants if they were ‘optimistic in terms of Afghanistan’s future,’ also with binary ‘yes-no’ response choice. Given their high correlation (*r* = 0.53), we created an index of hope and optimism by summing responses to these questions, scores of which range from 0 to 2 with higher scores indicative of higher perceived ‘hope-optimism’. With the exception of the ASCL, which was already available in the Dari language (provided courtesy of the scales developer—Dr. Ken Miller), all scales were translated from English to Dari using a rigorous translation-back-translation process with assistance from the Afghan refugee community in southern California.

### Data analysis

*SPSS*, *version 24.0* [23] was used for all data analyses. We evaluated standard assumptions of parametric tests, which warranted no data transformations; and, handled missing data by imputing the mean score for scales with less than 10% missing items [24]. Statistical significance was considered at *p* < .05 (though we indicate relationships approaching significance: *p* < .10). We calculated means and standard deviations along with percentages for variables where appropriate. Bivariate tests (independent samples *t*-tests, Pearson's '*r*') were used to identify variables significantly associated with our dependent variables, mental health status (MCS scores) and psychological distress (ASCL scores). Variables significantly associated with MCS and ASCL scores at the bivariate level were entered in two separate ordinary least square (OLS) regression models, one for MCS, and another for ASCL. To examine the unique contribution of each type of variable, socio-demographic factors were entered in Step 1 of each model (mainly consisting of risk-factors), followed by protective factors in subsequent steps). Unstandardized and standardized betas along with R^2^_*adjusted*_ and ΔR^2^ values are reported in Tables 3 and 4.

## Results

### Socio-demographic characteristics

Table 1 displays socio-demographic characteristics of our participants. Surveys were completed by 232 participants who were 23.4 (*SD* = 4.6) years of age. The majority were male (56.8%), unmarried (62.3%), of Pashtun ethnicity (36.8%), and unemployed (57.7%) with unstable incomes (63.6%). Unemployment rates observed here are considerably higher than general population rates of 46.2% reported in Kabul, whereas income instability rates mirror perceptions of worsening household financial conditions reported in 2016 [25]. Moreover, the majority indicated being both hopeful (57.7%) and optimistic (65%) about their own future in Afghanistan and their country’s socio-political future, respectively. CD-RISC-25 scores ranged from 26 to 92 with an average score of 60.5±13.9, which is approximately 20 points lower than the average score observed in a normative sample in the U.S. [18]. Mean PCS scores were reported at 45.6 (*SD* = 7.8). Further descriptive item responses show ‘role limitations due to physical health problems’ being rated lowest (43.6±8.9), followed by physical functioning (*M* = 44.1, *SD* = 8.3), perceived general health (*M* = 46.6, *SD* = 8.7), and lastly bodily pain (*M* = 50.3, *SD* = 9.2).

**Table 1:** Socio-demographic Characteristics (*N* = 232)

Variables	*n* (%)
Age: *M* = 23.36, *SD* = 4.64	–
Gender	
Female	90 (43.2)
Male	117(56.8)
Ethnicity	
Pashtun	73 (36.8)
Tajik	77 (35.9)
Other^a^	59 (27.3)
Marital Status	
Married	80 (38.7)
Not Married^b^	123 (62.3)
Education	
College and beyond	87 (40.3)
HS Diploma and lower	122 (59.7)
Employment	
Employed	87 (42.3)
Unemployed	118 (57.7)
Income	
Comfortable	76 (36.4)
Not comfortable	129 (63.6)
Hope w/re to own future	
Hopeful	119 (57.5)
Not Hopeful	88 (42.5)
Optimism w/re to Afg.’s Future	
Optimistic	134 (65.0)
Not Optimistic	72 (35.0)
CD-RISC-25: Resilience: *M* = 60.53, *SD* = 13.86	–
PCS Score: Physical Health Status: *M* = 45.59, *SD* = 7.77	–

^a^includes Hazaras, Nuristanis, Uzbeks; ^b^includes ‘never married’, and two participants widowed, and four participants divorced/separated

The mean MCS score was 42.5 (*SD* = 10.29), suggesting that participants generally have poor mental health when compared to the U.S. normative score of 50. Only 25% of our sample scored 50 or higher on the MCS. Further descriptive item responses show role limitations owing to personal or emotional problems seemed to affect participants most (*M* = 41.8, *SD* = 8.2), which was closely followed by mental health/emotional problems such as anxiety, depression, or irritability (*M* = 42.3, *SD* = 10.4); social functioning (*M* = 45.2, *SD* = 9.1) was also problematic, while vitality (*M* = 49.93, *SD* = 8.98) was rated highest. ASCL scores ranged from 23 to 94 with a mean of 47.6 (*SD* = 16.0). Some of the most frequently reported symptoms occurring at least once a week included ‘thinking too much’ (76%), ‘trouble concentrating’ (72%), ‘feeling sad’ (72%), becoming ‘jigar khun’ (70%), and headaches (69%).

### Bivariate analyses

Table 2 shows MCS and ASCL scores by our risk and protective factors. Poor mental health status and functional impairment (or lower MCS scores) is significantly associated with higher education, unemployment, and unstable income; whereas, higher physical health status, and being hopeful-optimistic are associated with higher MCS scores. The relationship between ethnicity and MCS scores was marginally significant with Tajiks reporting lower mental health status compared to Pashtuns and all other ethnicities.

**Table 2:** Bivariate Analyses

Variables	MCS Score	Statistic and *P*-value	ASCL Score	Statistic and P-value
	*M* (SD)		*M* (SD)	
Age	–	*r* = .070, *p* = .294	–	*r* = −.164, ***p*** **< .05**
Gender				
Female	42.32 (10.14)	*t*(223) = −1.51, *p* = .133	50.64 (14.96)	*t*(205) = 2.40, ***p*** **< .05**
Male	44.29 (9.36)		45.34 (16.31)	
Ethnicity				
Pashtun	44.06 (8.64)	*F*(2,224) = 2.92, *p* = .056	46.23 (15.01)	*F*(2,206) = 5.11**,** ***p*** **< .01**
Tajik	41.44 (10.98)		52.01 (16.75)	
Other^a^	45.19 (8.64)		43.75 (15.04)	
Marital Status				
Married	43.56 (9.41)	*t*(216) = −.21, *p* = .831	45.25 (14.04)	*t*(201) = 1.96, *p* = .05
Not Married^b^	43.27 (10.11)		49.56 (17.03)	
Education				
College and beyond	41.21 (10.24)	*t*(225) = 2.90, ***p*** **= .004**	50.93 (17.28)	*t*(207) = − 2.53, ***p*** **< .05**
HS Diploma and lower	44.96 (9.11)		45.33 (14.63)	
Employment				
Employed	45.28 (8.64)	*t*(221) = −2.61, ***p*** **= .010**	45.01 (11.90)	*t*(203) = 2.44, ***p*** **< .05**
Unemployed	41.96 (10.33)		50.15 (18.17)	
Income				
Stable	45.99 (9.29)	*t*(219) = 3.03, ***p*** **= .003**	43.71 (13.21)	*t*(203) = 3.03, ***p*** **< .01**
Unstable	41.91 (9.85)		50.18 (17.09)	
Hope-Optimism	–	*r* = .319*,* ***p*** **= .000**	–	*r* = −.279*,* ***p*** **< .000**
CD-RISC-25: Resilience	–	*r* = −.025*, p* = .732	–	*r* = −.040, *p* = .594
PCS Score: Physical Health Status	–	*r* = .376, ***p*** **= .000**	–	*r* = −.421, ***p*** **< .000**

^a^includes Hazaras, Nuristanis, and Uzbeks; ^b^includes ‘never married’, and two participants widowed, and four participants divorced/separated

However, when ethnicity was correlated with ASCL, we observed that Tajiks reported significantly higher psychological distress as compared to other ethnic groups. In addition, we observed that younger age and female gender were significantly associated with higher ASCL scores. Also, higher ASCL scores were associated with higher education, unemployment, unstable income; whereas lower were associated with good physical health and higher hope-optimism. Strikingly, our measure of resilience demonstrated a weak and non-significant relationship with both MCS and ASCL scores both in its full form and when the measure was divided according to the five constructs (personal competence, high standards, and tenacity; trust in one’s instincts, tolerance of negative affect, and strengthening effects of stress; positive acceptance of chance and secure relationships; control; and, spiritual influences). Therefore, the CD-RISC-25 was not included in subsequent (multivariable) analyses.

### Multivariable analyses

#### Factors associated with mental health status (MCS scores)

OLS regressions, shown in Table 3, indicate that demographic variables entered in Step 1 account for 11% of the variance in MCS scores with three variables significantly contributing to the model. Tajik ethnicity and the possession of a university degree are associated with poorer mental health status whereas stability in income is significantly associated with better mental health status. Only the significant effect of education remains in Step 2 after adding the physical health status variable to the model, which explains an additional 10% (ΔR^2^ = .101, *p* < .001) of the variance in MCS scores. There was a significant positive relationship between physical health status and MCS scores suggesting that higher physical health status is associated with higher mental health status. Interestingly, the beta for physical health demonstrates the strongest effect (*β* = 0.33, *p* < .001) on MCS scores and remains this way even after entering hope-optimism in Step 3. Though the hope-optimism variable adds significant variance to the model (ΔR^2^ = 0.05, *p* < .001 for a total of 27% of the variance explained) and shows that higher hope-optimism is associated with higher mental health status (*β* = 0.24, *p* < .001).

**Table 3:** OLS Regression Analysis of Factors Associated with Mental Health Status (MCS scores)

	Step 1^***^		Step 2^***^		Step 3^***^	
Variables	B (*SE*)	*β*	B (*SE*)	*β*	B (*SE*)	*β*
Age	.076 (.159)	.034	.068 (.150)	.031	.063 (.145)	.029
Gender: *female*^*a*^	−.908 (1.398)	−.046	−.417 (1.323)	−.021	−.430 (1.280)	−.022
Ethnicity: *Tajik*^*b*^	−3.06 (1.377)	−.149^*^	− 2.579 (1.303)	−.126^*†*^	− 2.353 (1.262)	−.115^*†*^
Education: *university degree*^*c*^	−2.96 (1.364)	−.147^*^	− 2.975 (1.287)	−.147^*^	−2.594 (1.249)	−.128^*^
Employment: *employed*^*d*^	2.312 (1.472)	.116	2.365 (1.389)	.118^*†*^	2.235 (1.344)	.112^*†*^
Income: *stable*^*e*^	3.985 (1.379)	.194^**^	2.132 (1.351)	.104	1.795 (1.310)	.087
Physical Health Status			.415 (.081)	.331^***^	.379 (.079)	.302^***^
Hope-Optimism					2.715 (.703)	.236^***^
*R* ^*2*^	.116		.216		.270	
Adjusted *R*^*2*^	.090		.189		.241	
Δ*R*^*2*^			.101^***^		.054^***^	
*F statistic* for change in *R*^*2*^			26.053		14.916	

N = 211 as 21 cases omitted by *listwise* deletion; Reference groups: ^a^males, ^b^Pashtuns and other ethnicities; ^c^HS Diploma, ^d^Unemployed, ^e^Unstable income; ^*†*^*p* < .10; *p < .05; **p < .01; ***p < .001

#### Factors associated with psychological distress symptoms (ASCL scores)

Table 4 shows the regression model for ASCL scores. Here, demographic variables in Step 1 account for approximately 17% of the variance in ASCL scores and parallel the MCS model, such that Tajiks, university graduates, and those reporting unstable incomes report significantly higher distress. In contrast to the MCS model, younger age and female gender approach significance with ASCL (*p* < .10). However, in Step 2 (younger) age emerges as significant, while no significant correlation was detected between gender and ASCL scores after entering the physical health status variable. Physical health status is inversely associated with psychological distress, and similar to the MCS model, possesses the highest beta (*β* = − 0.38, *p* < .001). In Step 3, hope-optimism adds significant variance to the overall model (ΔR^2^ = 0.04, *p* < .01) indicating that higher hope-optimism is associated with lower distress. In addition to hope-optimism, this full model shows that age, ethnicity, education, income, and physical health status contribute significantly to explaining ASCL scores (for a final 34% of variance explained).

**Table 4:** OLS Regression Analysis of Factors Associated with Psychological Distress Symptoms (ASCL scores)

	Step 1^***^		Step 2^***^		Step 3^***^	
Variables	B (*SE*)	*β*	B (*SE*)	*β*	B (*SE*)	*β*
Age	−.522 (.265)	−.139^*†*^	−.550 (.244)	−.156^*^	−.537 (.237)	−.152^*^
Gender: *female*^*a*^	−2.857 (2.34)	.138^*†*^	−1.498 (2.17)	−.046	−1.586 (2.11)	−.049
Ethnicity: *Tajik*^*b*^	6.507 (2.27)	.196^**^	5.008 (2.11)	.150^*^	4.835 (2.05)	.145^*^
Education: *university degree*^*c*^	5.199 (2.27)	.147^*^	6.140 (2.10)	.186^**^	5.616 (2.05)	.170^**^
Employment: *employed*^*d*^	−.911 (2.46)	.024	−.649 (2.27)	−.020	−.585 (2.21)	−.018
Income: *stable*^*e*^	−7.872 (2.28)	−.236^**^	−4.805 (2.17)	−.143^*^	−4.292 (2.12)	−.128^*^
Physical Health Status			−.785 (.135)	−.375^***^	−.723 (.133)	−.346^***^
Hope-Optimism					−3.910 (1.15)	−.208^**^
*R* ^*2*^	.167		.295		.337	
Adjusted *R*^*2*^	.140		.268		.308	
Δ*R*^*2*^			.128^***^		.042^**^	
*F statistic* for change in *R*^*2*^			33.765		11.665	

N = 197 as 35 cases omitted by *listwise* deletion; Reference groups: ^a^males, ^b^Pashtuns and other ethnicities; ^c^HS Diploma, ^d^Unemployed, ^e^Unstable income; ^*†*^*p* < .10; ^*^p < .05; ^**^p < .01; ^***^p < .001

## Discussion

We conducted a community-based cross-sectional study examining mental health and psychological distress symptoms among young adults residing in Kabul in terms of poor social conditions, rather than primarily war-trauma induced distress in keeping with the findings of Miller and Rasmussen’s [7] work. In using two distinct scales, our work shows that the mental health status of young adults is poor when compared to U.S. normative scores, but comparable to other conflict-affected groups such as northern Ugandans [21]. And based on the ASCL, we also found that many endure a number of depressive, anxiety-like, and somatoform symptoms. It is noteworthy that both multivariate models were able to explain high degree of variance with the variables we selected. Our first hypothesis related to demographic variables is partially supported. As expected, we observed that both MCS and ASCL scores were influenced by an array of social factors, namely– ethnicity, unstable income and higher education, the latter consistently maintaining a significant relationship with poorer outcomes in each multivariate model. This relationship reflects the pervasive disillusionment that a broken economy has given rise to, one which does not provide jobs for the surplus of university graduates. This aligns with Panter-Brick et al.’s [11] observations of educated, young adults in Kabul.

The hypothesis related to demographic variables was shown more strongly in the ASCL scale than the MCS scale. Younger age only contributed to the ASCL model (potentially highlighting a stage of life uncertainty and lack of opportunity). Importantly, women, while shown to be more distressed in bivariate analyses, were no more likely than men to report poorer outcomes in multivariate analyses, which does not support our hypothesis and contrasts with prior research in Afghanistan [6, 11]. Men and women inhabit unique social and emotional spaces in Afghanistan, according to Rasmussen et al. [16], and likely perceive and experience stress differently partly due to men’s higher levels of social interaction in the public sphere. Therefore, observing this non-significant relationship was surprising, especially in light of using the ASCL, a culturally-salient scale able to tap into locally-relevant dimensions of mental health that may be more salient for women (e.g. maintaining harmonious family relationships) [15], which we would have expected to more likely be able to identify distress, if it was indeed different from patterns seen in men. Nevertheless, these results extend our knowledge of the impact that daily stressors have on mental health, as originally hypothesized by Miller et al. [6], and to some degree coincides with mental health risk-factors reported in a systematic review of conflict-affected populations in low- and middle-income countries [26].

The correlation between Tajik ethnicity and poor outcomes is noteworthy, particularly in the ASCL model. Similarly, in their household survey, Scholte et al. [5] found that PTSD symptomatology was higher among Tajiks as compared to other ethnicities, but provide no further insight as to why. We speculate that their perceived stress may be a result of feeling disadvantaged or marginalized in a Pashtun-dominated society, or perhaps that Pashtuns may be more guarded and less expressive of their disclosure of symptoms, though further research is needed to replicate and better understand this pattern.

Moreover, the physical health status of our participants was poor when compared to U.S. normative scores while being comparable to PCS scores observed among conflict-affected Ugandans [21]. PCS scores were significantly associated with both the MCS and ASCL models, having a protective effect on mental health and distress symptoms. This result is reasonable given that physical health is widely understood to be significantly related to mental health. Strikingly, our hope-optimism variable emerged as an important coping mechanism in this context, so much so that it added substantial explained variance when added last to both MCS (5.4%) and ASCL (4.2%) models. The concept of hope, according to Eggerman and Panter-Brick’s [27] qualitative study is a critical component of resilience in Afghanistan, which is rooted in moral and social order embodied through the expression of Afghan cultural values that include their (Islamic) faith, family unity, service to society, effort, moral behavior, and upholding family honor. In considering this, we assert that our measure of hope-optimism likely provides an indication of “collective” resilience, which explains why higher hope-optimism here is associated with improved mental health status and lower distress symptoms.

This also explains why scales assessing “individual resilience” such as the CD-RISC-25, used here, may lack content validity in a collectivistic society, in which individuals possess a communal sense of self and view themselves inseparable from the social context [28]. The lack of correlation between our hope-optimism variable and our measure of resilience further validates our position. We noticed that our CD-RISC-25 scores ranged from 26 to 92 with an average score of 60.53 (*SD* = 13.86). This is approximately 20 points lower than the average score observed in normative U.S. samples [22, 29]. Our findings on CD-RISC are congruent with studies of other non-Western, collectivistic cultures: 66.8 mean score in Korea [30], 65.4 in China [31], and 60.0 in Hong Kong [32]. Interestingly, a CD-RISC-25 survey [33] of resilience in refugees from Africa (mean 60), Middle East (mean 67), and former Yugoslavia (mean 69) suggests a linear distribution of the resilience scores along a cultural spectrum between individualistic cultures at one end, and collectivistic cultures, at the other, as hypothesized in a 2011 Kyrgyz study [34]. Importantly, this Kyrgyz study found that the correlation between measures of PTSD and an abridged version of the CD-RISC were poor. The authors infer that one reason for the associations between CD-RISC and distress could be a cultural difference in item interpretation and test settings [35]. This coincides with Suarez’ [36] results using this measure of resilience alongside cultural idioms of distress in a sample of conflict-affected Peruvian girls, where resilience was found to be more deeply rooted in community and cultural realms and CD-RISC scores were not associated with lower levels of distress.

This suggests that risk and individual resiliency cannot be understood apart from context; self-conceptualizing in relationship to traumatic experiences varies widely across cultures constructing resilience as a cultural phenomenon [37]. Indeed, communities are vital sources of resilience by making needed resources available for individuals with much needed material, emotional, and cognitive reframing support. However, when traumatic events disrupt these vital support networks at most critical times, communities can actually add more turmoil and distress [38]. Therefore, future Kyrgyz, or in our case, Afghan studies, should investigate more closely the relationship between community and individual resilience, and develop community resilience instruments that are culturally-grounded using inductive techniques. This could have important clinical applications in community psychiatry interventions to shift the current traditionally targets of cognitive-behavior, and cognitive-processing therapies for disorders such as PTSD, from “I” and “self-image”, to “Us”. Such instruments could then be applied to evaluations of interventions that consider an array of psychosocial factors, especially building one’s capacity to access resources needed to create social and economic opportunities for one’s family [39].

Moreover, public health professionals and policy makers alike should consider how such interventions ought to be delivered. Patel et al. [12] suggests that interventions (reducing risk-factors and strengthening protective factors) could be disseminated through community-based channels, educational settings such as colleges, the internet, and through trained primary care practitioners – the latter being a viable approach given that public mental health services have been formally integrated into the primary care sector in Afghanistan [40]. Additionally, given the highly somatic nature of distress in our sample, the primary care sector provides a realistic avenue for help seeking in a society where mental health problems are highly stigmatized. In addition to formal health services, interventions could be delivered through the informal care sector, perhaps through clergy and herbalists, both of which young people may be inclined to seek help from, which is an area that our future work will concentrate on.

Our work clearly has some limitations. The cross-sectional design does not allow for causal inferences, and there may have been a potential for selection bias due to the convenience sampling technique used. Also, the present study does not adjust for exposure to traumatic experiences, which may have influenced our findings. Another important factor not adjusted for here is one’s engagement in civic life or political activity, which has been shown to be both protective and promotive in several studies of conflict-affected children in low- and middle-income countries [41]. Nevertheless, the strength of this contribution lies in the use of etic and emic scales for mental health and psychological distress, respectively, and the fact that our sample represents wide variation across a number of socio- demographic and -economic characteristics, lending credence to the generalizability of our findings within urban Kabul and perhaps other urban regions of Afghanistan, but certainly not to other social and cultural groups outside the country.

## Conclusions

In conclusion, our research shows that young adults in Kabul present with poor mental health status and distress symptoms as a result of a number of social factors rooted in the country’s current conditions. The evidence from this study suggests that while mental health problems are widely present, possessing good physical health and hope-optimism serve as protective factors. This has important implications for tailoring context-specific policy and programmatic interventions for young adults. Interventions could, at least in part, focus on addressing the less stigmatizing issues young people may face related to physical health, while building up hope and optimism. To support the physical health of young people and their aspirations for realizing a better future, policy and structural changes such as improving access to employment are important to not only strengthen the economy and their living conditions, but to help them deal with their current distress and social functioning issues. Interventions aimed at improving hope and optimism could very well be community based and include aspects of Afghan cultural values related to strengthening family unity and faith, serving society, as well as maintaining moral behavior and upholding honor [27].

## Abbreviations

ASCL: Afghan symptom checklist
CD-RISC-25: Connor-davidson resilience scale
MCS: Mental component summary
OLS: Ordinary least squares
PCS: Physical component summary
PTSD: Post-traumatic stress disorder
SF-8: Short-form
U.S.: United States
